# Diabetes mellitus in stable chronic heart failure and the combination with humoral activation, their association, and prediction of 2‐year adverse outcomes. Data from the FAR NHL registry

**DOI:** 10.1111/1753-0407.13605

**Published:** 2024-09-12

**Authors:** Karel Labr, Jindrich Spinar, Jiri Parenica, Lenka Spinarova, Jan Krejci, Filip Malek, Petr Ostadal, Ondrej Ludka, Jiri Jarkovsky, Klara Benesova, Ruzena Labrova, Monika Spinarova

**Affiliations:** ^1^ First Department of Internal Medicine – Cardioangiology St. Anne's University Hospital, Faculty of Medicine, Masaryk University Brno Czech Republic; ^2^ Department of Internal Cardiology Medicine University Hospital Brno, Faculty of Medicine, Masaryk University Brno Czech Republic; ^3^ Department of Cardiology Na Homolce Hospital Prague Czech Republic; ^4^ Department of Cardiology, 2nd Medical Faculty Charles University and University Hospital Motol Prague Czech Republic; ^5^ Department of Internal Medicine, Geriatrics and Practical Medicine University Hospital Brno, Faculty of Medicine, Masaryk University Brno Czech Republic; ^6^ Institute of Biostatistics and Analyses, Masaryk University Brno Czech Republic

**Keywords:** chronic heart failure, diabetes mellitus, N‐terminal pro‐brain natriuretic peptide, prognosis

## Abstract

**Background/Aim:**

The study aims to describe the role of diabetes in patients with heart failure.

**Methods:**

In all, 1052 chronic heart failure patients were included in the FARmacology and NeuroHumoral Activation (FAR NHL) multicenter prospective registry. They had ejection fraction below 50% and were on stable medication for at least 1 month.

**Results:**

More than one‐third (38.9%) of the patients had diabetes mellitus (DM). Diabetic patients (*N* = 409) were older (median 67 vs. 64, *p* < 0.001), had higher body mass index (BMI) (30 vs. 28 kg/m^2^, *p* < 0.001), much more frequently had ischemic heart disease (71 vs. 47%, *p* < 0.001), hypertension (80 vs. 67%, *p* < 0.001), dyslipidemia (89 vs. 69%, *p* < 0.001), worse renal function (estimated glomerular filtration rate [eGFR] median 63 vs. 73 mL/min/1.73 m^2^, *p* < 0.001), and higher N‐terminal pro‐brain natriuretic peptide (NT‐proBNP) (median 681 vs. 463 pg/mL, *p* = 0.003). All‐cause death, left ventricle assist device implantation, and orthotopic heart transplantation were set as the combined primary end point, which was present in 15.5% (163 patients) within the 2‐year follow‐up. In the 2‐year follow‐up, 81.0% of patients with diabetes survived without a primary end point, while 85.4% of the patients without diabetes survived, the difference being on the verge of statistical significance (*p* = 0.089). DM is a statistically significant predictor of NT‐proBNP value in univariate analysis, but it is not an independent predictor in a multivariate analysis. When the NT‐proBNP level was high, the presence of DM did not influence the prognosis.

**Conclusion:**

The combination of diabetes and NT‐proBNP levels may better stratify the prognosis of patients with chronic heart failure.

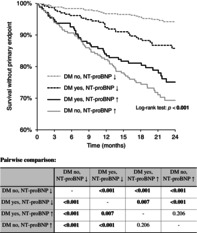

## Graphical abstract

Highlights

- Diabetic patients more often has comorbidities such as ischemic heart disease, hypertension, dyslipidemia, or renal impairment, higher uric acid, lower hemoglobin, and lower eGFR.
- Based on a large prospective multicenter registry, we found that the combination of diabetes and NT‐proBNP levels may help stratify the prognosis of patients with chronic heart failure when the NT‐proBNP level is low.
- High NT‐proBNP is in itself a strong predictor of events (all‐cause death, left ventricle assist device implantation, and orthotopic heart transplantation), that the addition of diabetes mellitus will no longer improve the stratification of patient prognosis in this group.

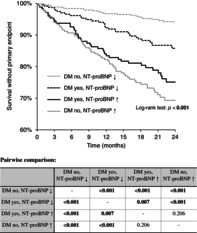

## 1 INTRODUCTION

Diabetes mellitus (DM) is a very important comorbidity of heart failure (HF) and is associated with worse functional status and prognosis, which was already described in the Framingham study.^1^ The two diseases are closely intertwined, and worsening of one condition is often accompanied by worsening of the other.^2^, ^3^ The prevalence of patients with concomitant HF and DM is high. A large European‐wide registry found that 36% of patients with stable cardiovascular (CV) disease had diabetes, while up to 50% of patients hospitalized for acute CV disease requiring intravenous CV therapy had diabetes.^4^ Patients with HF but no diabetes have an increased risk of developing DM, and this risk increases with the severity of CV disease and the use of loop diuretics.^5^ The prevalence of DM is increasing rapidly with increasing rates of obesity, population aging, and other risk factors.^6^ Diabetes is associated with a higher risk of CV disease and death. Determining and stratifying the prognosis of patients with HF helps patients, their families, and physicians to determine and optimize the timing of treatment and can also assist in planning for future demands on health and social services. There are many prognostic markers for HF. Therefore, more research is needed to describe this population in each region.

The work from Bhalla et al. revealed that B‐type natriuretic peptide appears to be a reliable predictor of future cardiac and all‐cause mortality in diabetic patients, but without considering the severity of HF.^7^ In the Chinese study by Peng et al., the plasma BNP level was significantly higher in the DM group than in the non‐DM group at the same HF score.^8^ The study from Fringu et al. has shown that there were no significant differences between the mean N‐terminal pro‐brain natriuretic peptide (NT‐proBNP) values in patients with or without DM; however, this biomarker still holds a pivotal role in the evaluation of subjects with HF and DM, facilitating the establishment of correct management and follow‐up.^9^

We therefore set out to describe the role of diabetes in patients with HF in the Czech Republic and its impact on prognosis, including its association with natriuretic peptides. We hypothesize that the combination of NT‐proBNP and DM could lead to a better stratification of patients with systolic chronic HF.

## 2 METHODS

### 2.1 Data collection and analysis

The data come from a multicenter prospective registry, FARmacology and NeuroHumoral Activation (FAR NHL), collected from October 1, 2014 to November 30, 2015, in three cardiology centers in the Czech Republic specializing in HF (First Department of Internal Medicine—Cardioangiology, St. Anne's University Hospital in Brno; Department of Internal Cardiology Medicine, University Hospital Brno; and Department of Cardiology, Na Homolce Hospital, Prague). FAR NHL registry is a database of patients with stable systolic chronic HF. Patients had to be treated for HF with nonpreserved ejection fraction (EF <50%) and had to be stable for at least 1 month with optimal pharmacotherapy in order to be included in the registry. The aim of this registry was to describe the HF population of patients, their pharmacotherapy, both standard and new humoral substances, and markers.

All patients signed informed consent. The study was conducted according to the guidelines of the Declaration of Helsinki and approved by the Ethics Committee of Brno University Hospital, Czech Republic (protocol code 02‐221014/EK, date of approval 22 October 2014).

The study included stable outpatients and hospitalized patients with chronic HF in whom the reason for hospitalization was not only acute HF (AHF) but also cardiac status examination before consideration of possible orthotopic heart transplantation (OHT) or left ventricular assist device (LVAD) implantation. In the FAR NHL registry patients had stable chronic HF for at least 30 days and were with HF with mid‐ranged and reduced left ventricular EF (LVEF) below 50%. Patients who had undergone OHT or LVAD implantation were initially excluded. We examined age, sex, anamnestic data, and comorbidities and clinical examination including blood pressure and ECG in these patients. In addition, blood samples were taken, and basic biochemical and hematological analyses were performed, including the determination of NT‐proBNP. In the FAR NHL registry, more humoral agents were evaluated and published.^10^, ^11^, ^12^, ^13^, ^14^ The medication used was also processed.^15^, ^16^, ^17^

Follow‐up was conducted as of December 31, 2016, from the hospital information system, the database of the State Institute for Drug Control, and by telephone directly from patients. Follow‐up was focused on deaths and LVAD implants or OHT. All patients had data for at least 1 year from enrolment. For some patients enrolled in previous months, data were available from up to 2 years of follow‐up.

The combined primary end point was defined as death from all causes, LVAD implantation, or heart transplantation during follow‐up.

Out of 1095 valid records in the FAR NHL database, NT‐proBNP values were unavailable for 36 patients, and follow‐up data for 7 patients were not obtained. The remaining 1052 patients were enrolled in this study. This group was divided into two subpopulations—patients with and without diabetes. Criteria for inclusion in the diabetes group were self‐reported physician‐diagnosed DM (types 1 and 2) regardless of the duration of the condition, reported in the documentation, or use of hypoglycemic medication (insulin or oral antidiabetics). Both subgroups underwent the same examinations. The cutoff value of NT‐proBNP was determined by receiver operating characteristic analysis at 1000 pg/mL.

Plasma levels of NT‐proBNP were analyzed by electrochemiluminescence at all three centers using a Cobas analyzer for the NT‐proBNP Immunoassay Kit (Roche Diagnostics, Indianapolis, IN, USA). The detection limit of NT‐proBNP was 5 pg/mL, and the calibration range was 5–35 000 pg/mL. The coefficient of variation in the centers ranged between 1.4% and 5.8%.

### 2.2 Statistical analysis

The data were processed by the statisticians from the Institute of Biostatistics and Analyses (Masaryk University, Brno, Czech Republic). Standard descriptive statistics were applied in the analysis; continuous variables were described by mean ± SD or, in case of laboratory results, by median and interquartile range, whereas categorical variables were characterized by absolute and relative frequencies. Statistical significance of differences among groups of patients was analyzed using the Mann–Whitney test or the Kruskal–Wallis test for continuous variables and the Fisher's exact test for categorical variables. The Kaplan–Meier methodology was used to visualize survival data, and the statistical significance of differences in survival between patient groups was assessed using a log‐rank test. As a level of statistical significance, *p* = 0.05 was used in all analyses. Analysis was performed in SPSS 28.0.1.1 (IBM Corporation, Armonk, NY, USA, 2021).

## 3 RESULTS

### 3.1 Baseline characteristics

A total of 1052 patients with stable HF with mid‐ranged and reduced LVEF were enrolled; 81% were male, and the median age was 65 years. The etiology of HF was ischemic heart disease in 49.4% of the patients, dilated cardiomyopathy in 42.3%, and hypertrophy cardiomyopathy in 0.5%. Most of the patients (69%) were in class II of the New York Heart Association classification (NYHA). The median of blood pressure was 127/79 mm Hg, and the median of heart rate was 72 bpm.

Overall, 409 HF patients (38.9%) had DM. Diabetic patients were older (median 67 [60–73] vs. 64 [54–72] years; *p* < 0.001), had higher body mass index (BMI) (30 [27–34] vs. 28 [25–31] kg/m^2^; *p* < 0.001), much more frequently had ischemic heart disease (71 vs. 47%, *p* < 0.001), hypertension (80 vs. 67%, *p* < 0.001), dyslipidemia (89 vs. 69%, *p* < 0.001), worse renal function (estimated glomerular filtration rate [eGFR] 63 [47–82] vs. 73 [57–89] mL/min/1.73 m^2^, *p* < 0.001), and higher NT‐proBNP (681 [225–1078] vs. 463 [138–1462] pg/mL, *p* = 0.003). Diabetic patients more frequently had leg swellings (26.7 vs. 15.7%; *p* < 0.001). There were no significant differences in blood pressure (systolic 129 vs. 128; *p* = 0.983), EF (30% in both groups, *p* = 0.152), or the presence of atrial fibrillation (13.9 vs. 12.3, *p* = 0.452). Furosemide was more frequently administered to the diabetic group (86 vs. 77%, *p* < 0.001). The incidence of other main drugs for HF was similar in both groups: angiotensin‐converting enzyme inhibitors (ACEi) or angiotensin receptor blockers (ARBs) (86.6% vs. 89.3%, *p* = 0.202), beta‐blockers (BB) (93.6% in both groups, *p* = 1.000), or mineralocorticoid receptor antagonists (MRA) (68.0 vs. 63.9, *p* = 0.184). The baseline characteristics of the groups are shown in Table 1.

**TABLE 1:** Characteristics of patients with chronic heart failure according to the presence of diabetes mellitus (DM).

	DM, yes (*N* = 409)	DM, no (*N* = 643)	*p*‐Value
Basic characteristics			
Age	67 (60–73)	64 (54–72)	**<0.001**
Sex: men	337 (82.4%)	513 (79.8%)	0.335
BMI (kg/m^2^)	30 (27–34)	28 (25–31)	**<0.001**
Systolic BP (mmHg)	129 (120–138)	128 (116–140)	0.983
Heart rate (bpm)	74 (65–81)	71 (64–80)	**0.010**
Leg swelling	109 (26.7%)	101 (15.7%)	**<0.001**
Ischemic heart disease	290 (70.9%)	304 (47.3%)	**<0.001**
Hypertension	326 (79.7%)	359 (55.8%)	**<0.001**
Dyslipidemia	364 (89.0%)	445 (69.2%)	**<0.001**
Ejection fraction	30 (25–35)	30 (25–38)	0.152
Atrial fibrillation	57 (13.9%)	79 (12.3%)	0.452
Diastolic dysfunction	248 (60.6%)	356 (55.4%)	0.097
Uric acid (μmol/L)	412 (351–477)	388 (330–463)	**0.003**
Hemoglobin (g/L)	141 (130–151)	145 (133–154)	**<0.001**
eGFR (mL/min/1.73 m^2^)	63 (47–82)	73 (57–89)	**<0.001**
NT‐proBNP (pg/mL)	681 (225–1708)	463 (138–1462)	**0.003**
Medication			
ACEi/ARB	354 (86.6%)	574 (89.3%)	0.202
Beta‐blockers	383 (93.6%)	602 (93.6%)	1.000
Furosemide	351 (85.8%)	497 (77.3%)	**<0.001**
MRA	278 (68.0%)	411 (63.9%)	0.184

*Note*: Continuous variables are described by median (interquartile range [IQR]); categorical variables are described by absolute and relative frequencies. *p*‐value of Mann–Whitney *U* test for continuous variables and *p*‐value of the Fisher's exact test for categorical variables are reported for comparison of patients' characteristics according to the presence of DM. Bold indicates statistically significant values.

Abbreviations: ACEi, angiotensin‐converting enzyme inhibitors; ARB, angiotensin receptor blockers; BMI, body mass index; BP, blood pressure, eGFR, estimated glomerular filtration rate; MRA, mineralocorticoid receptor antagonists; NT‐proBNP, N‐terminal pro‐brain natriuretic peptide.

### 3.2 NT‐proBNP values

**FIGURE 1:**
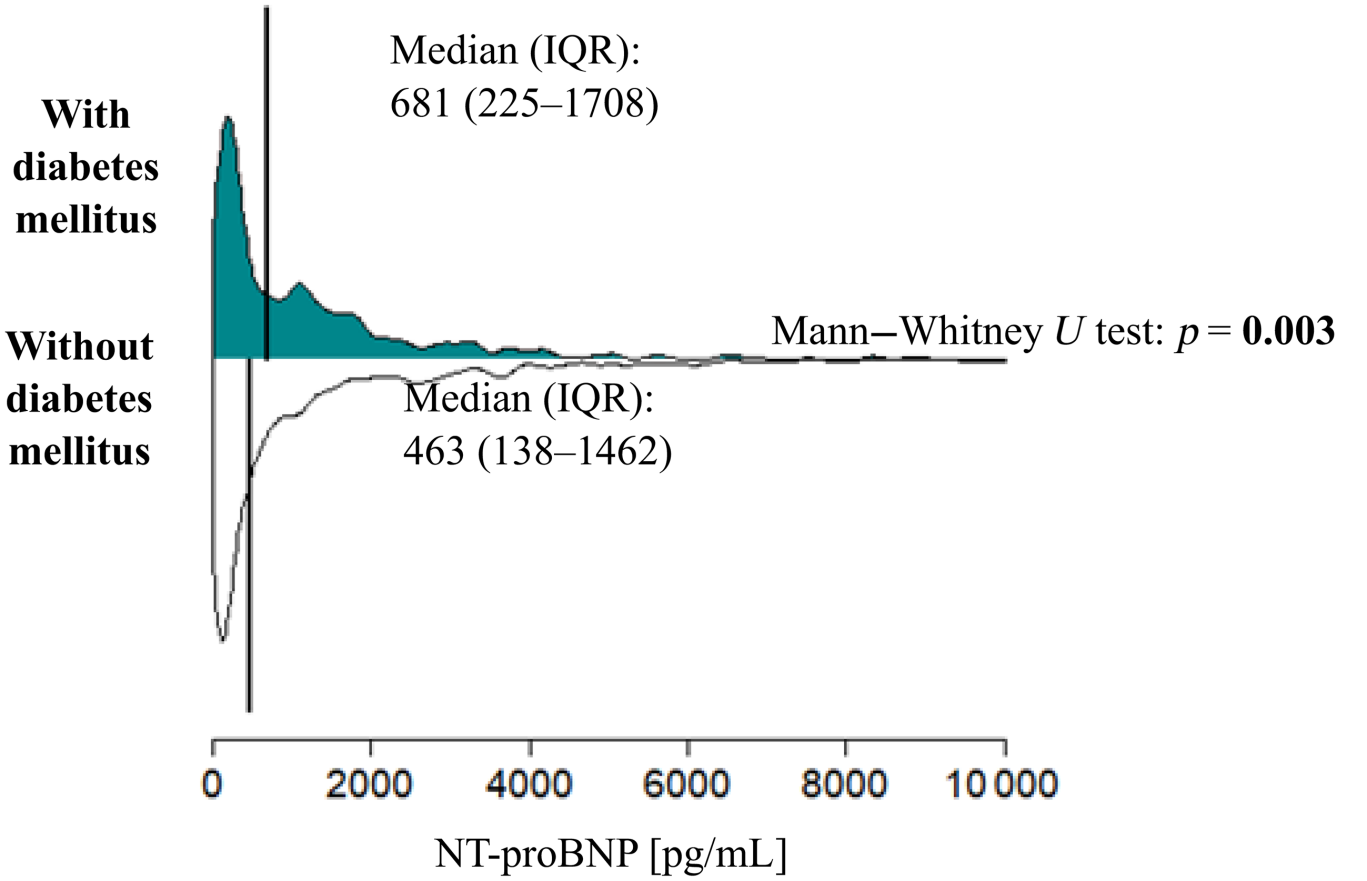
Distribution of N‐terminal pro‐brain natriuretic peptide (NT‐proBNP) in patients with and without diabetes mellitus. IQR, interquartile range.

Patients with DM had much higher NT‐proBNP (681 [225–1078] vs. 463 [138–1462] pg/mL, *p* = 0.003). The distribution of NT‐proBNP in both groups is shown in Figure 1.

Diabetes is statistically significantly associated with higher NT‐proBNP levels in univariate analysis (Table 2), but not in multivariate analysis (Table 3). Diabetic patients more often have comorbidities such as ischemic heart disease, hypertension, dyslipidemia, or renal impairment, higher uric acid, lower hemoglobin, and lower eGFR. These parameters are associated with higher NT‐proBNP values. Higher NT‐proBNP in diabetic patients are explained by these factors, not the very presence of diabetes.

**TABLE 2:** Univariate linear regression models for prediction of NT‐proBNP value.

Predictor	Change	exp(*β*) (95% CI)	*p*‐Value	*R* ^2^
Diabetes mellitus	Yes (ref. No)	1.35 (1.11–1.64)	**0.003**	0.8%
Age	10‐unit increase	1.19 (1.10–1.28)	**<0.001**	1.7%
Sex	Men (ref. Women)	1.04 (0.81–1.32)	0.773	0.0%
BMI	1‐unit increase	0.93 (0.92–0.95)	**<0.001**	4.5%
Systolic BP (mmHg)	10‐unit increase	0.79 (0.75–0.83)	**<0.001**	6.7%
Heart rate (bpm)	10‐unit increase	1.24 (1.16–1.34)	**<0.001**	3.1%
Leg swelling	Yes (ref. No)	1.03 (0.81–1.31)	0.814	0.0%
Ischemic heart disease	Yes (ref. No)	1.25 (1.03–1.52)	**0.022**	0.5%
Hypertension	Yes (ref. No)	1.13 (0.92–1.39)	0.230	0.1%
Dyslipidemia	Yes (ref. No)	1.01 (0.80–1.27)	0.935	0.0%
Ejection fraction	5‐unit increase	0.70 (0.66–0.73)	**<0.001**	16.4%
Fibrillation	Yes (ref. No)	2.29 (1.72–3.03)	**<0.001**	3.0%
Diastolic dysfunction	Yes (ref. No)	4.36 (3.67–5.19)	**<0.001**	21.0%
Uric acid (μmol/L)	100‐unit increase	1.45 (1.33–1.59)	**<0.001**	6.4%
Hemoglobin (g/L)	10‐unit increase	0.81 (0.77–0.86)	**<0.001**	4.1%
eGFR (mL/min/1.73 m^2^)	10‐unit increase	0.83 (0.80–0.87)	**<0.001**	6.7%
ACEi/ARB	Yes (ref. No)	0.62 (0.46–0.84)	**0.002**	0.9%
Beta‐blockers	Yes (ref. No)	0.70 (0.47–1.04)	0.078	0.3%
Furosemide	Yes (ref. No)	2.07 (1.63–2.63)	**<0.001**	3.3%
Verospiron	Yes (ref. No)	1.18 (0.97–1.45)	0.104	0.3%

*Note*: Continuous variables are described by median (interquartile range [IQR]); categorical variables are described by absolute and relative frequencies. *p*‐value of Mann–Whitney *U* test for continuous variables and *p*‐value of the Fisher's exact test for categorical variables are reported for comparison of patients' characteristics according to the presence of diabetes mellitus. Bold indicates statistically significant values.

Abbreviations: ACEi, angiotensin‐converting enzyme inhibitors; ARB, angiotensin receptor blockers; BMI, body mass index; BP, blood pressure; CI, confidence interval; eGFR, estimated glomerular filtration rate; NT‐proBNP, N‐terminal pro‐brain natriuretic peptide.

**TABLE 3:** Multivariate linear regression model using backward stepwise algorithm for selection of independent predictors of NT‐proBNP value.

Predictor	Change	exp(*β*) (95% CI)	*p*‐Value
BMI	1‐unit increase	0.95 (0.93–0.96)	**<0.001**
Systolic BP (mmHg)	10‐unit increase	0.95 (0.92–1.00)	**0.029**
Heart rate (bpm)	10‐unit increase	1.14 (1.07–1.20)	**<0.001**
Ischemic heart disease	Yes (ref. No)	1.24 (1.08–1.43)	**0.003**
Ejection fraction	5‐unit increase	0.75 (0.72–0.78)	**<0.001**
Fibrillation	Yes (ref. No)	2.18 (1.76–2.69)	**<0.001**
Diastolic dysfunction	Yes (ref. No)	4.03 (3.51–4.63)	**<0.001**
Uric acid (μmol/L)	100‐unit increase	1.16 (1.08–1.24)	**<0.001**
Hemoglobin (g/L)	10‐unit increase	0.87 (0.83–0.91)	**<0.001**
eGFR (mL/min/1.73 m^2^)	10‐unit increase	0.89 (0.86–0.92)	**<0.001**

*Note*: Continuous variables are described by median (interquartile range [IQR]); categorical variables are described by absolute and relative frequencies. *p*‐value of Mann–Whitney *U* test for continuous variables and *p*‐value of the Fisher's exact test for categorical variables are reported for comparison of patients' characteristics according to the presence of diabetes mellitus. Bold indicates statistically significant values.

Abbreviations: BMI, body mass index; BP, blood pressure; CI, confidence interval; eGFR, estimated glomerular filtration rate; NT‐proBNP, N‐terminal pro‐brain natriuretic peptide.

### 3.3 Follow‐up data

**FIGURE 2:**
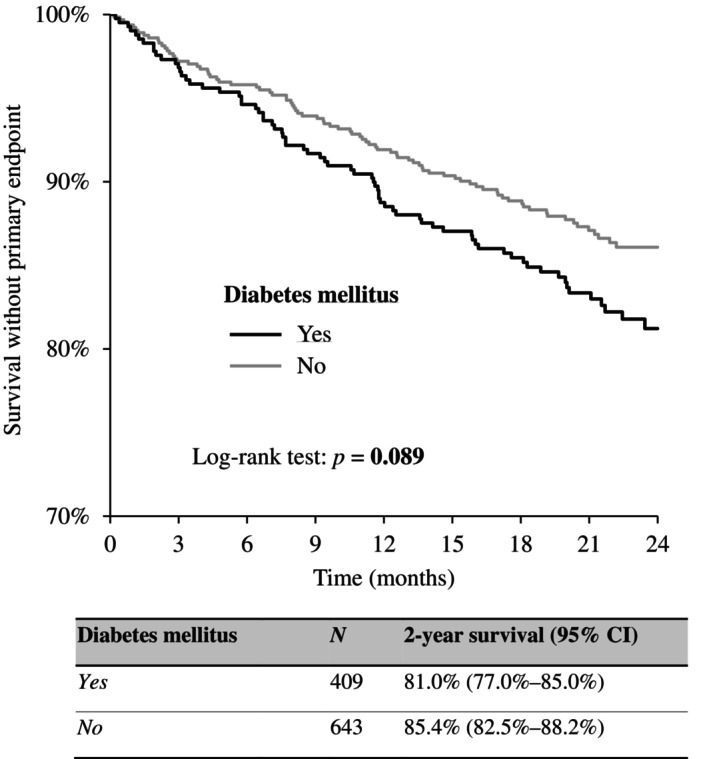
Two‐year survival without primary end point (all‐cause death, left ventricular assist device [LVAD], orthotopic heart transplantation [OHT]) in patients with chronic heart failure according to the presence of diabetes mellitus.

All‐cause death, left ventricle assist device implantation, and OHT were set as the combined primary end point, which was present in 15.5% of the patients (163 patients). At 2‐year follow‐up, 81.0% of patients with diabetes survived without the primary end point, while 85.4% of patients without diabetes survived, a difference that was already beyond statistical significance (*p* = 0.089). The data are shown in Figure 2.

**FIGURE 3:**
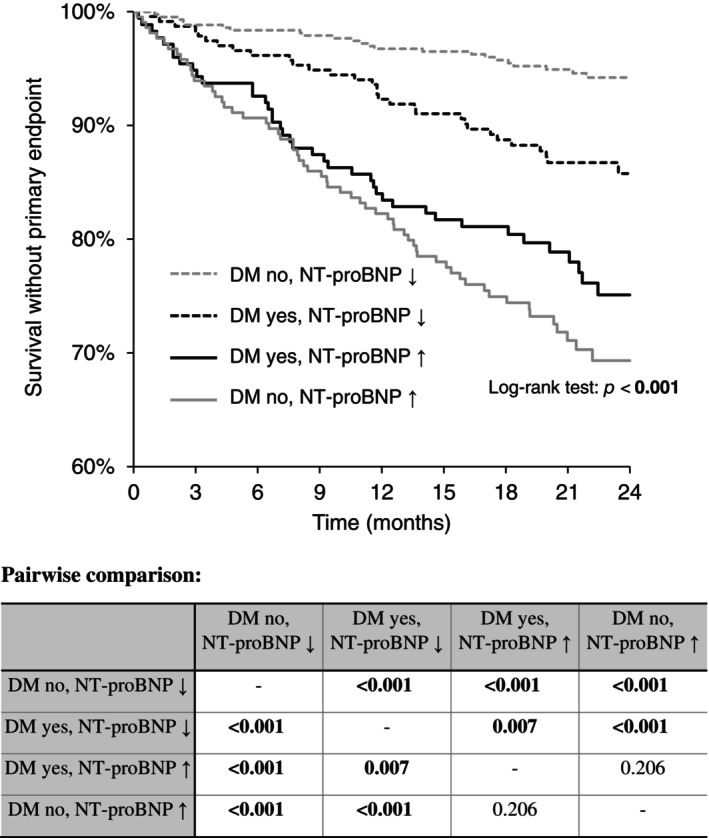
Two‐year survival without primary end point (all‐cause death, left ventricular assist device [LVAD], orthotopic heart transplantation [OHT]) in patients with chronic heart failure according to the presence of diabetes mellitus (DM) and N‐terminal pro‐brain natriuretic peptide (NT‐proBNP) value (<1000 vs. ≥1000 pg/mL).

A combination of DM and NT‐proBNP was subsequently used to stratify patients' prognoses. The highest 2‐year event‐free survival was reported in patients without diabetes and with low NT‐proBNP, followed by DM patients with low NT‐proBNP levels, then followed by DM patients with high NT‐proBNP, and patients without diabetes but with high levels of NT‐proBNP. The differences between the groups were statistically significant except for the last two groups (*p* = 0.160). When the NT‐proBNP level was high, the existence of DM did not influence the prognosis. The graph and pairwise comparison table are shown in Figure 3.

## 4 DISCUSSION

This article summarizes the baseline characteristics of HF patients with mid‐ranged and reduced EF from three teaching hospitals in the Czech Republic and the follow‐up data collected within 1 year of recruitment, so that for some patients, data were available for up to 2 years of follow‐up from the start of recruitment. We demonstrated the added value of the presence of DM to the NT‐proBNP value in predicting the prognosis of patients, especially when NT‐proBNP is low. Furthermore, we compared patients with stable HF with and without DM in relation to the presence of comorbidities and laboratory markers.

Furosemide was more frequently administered in the diabetic group. The prevalence of other major HF medications was similar in the two groups, with ACEi or ARBs, BB, or MRA, and was comparable. Compared with other registries, for example, the national HF registry in the Czech Republic, our patients received HF medications more frequently; for example, BB in 93.6% versus 82.1% and ACEi or ARBs in 88.4% versus 81.6% of the patients.^18^, ^19^ At the time of patient recruitment, angiotensin receptor‐neprilysin inhibitor (ARNI) or sodium–glucose cotransporter 2 inhibitor (SGLT2i) drugs were not yet routinely administered.

In the new 2023 European Society of Cardiology (ESC) Guidelines for the management of CV disease in patients with diabetes, the management of HF with reduced or mildly reduced EF remains the same regardless of the presence of diabetes: every patient should receive ACEi or ARNI, BB, MRA, and SGLT2i.^20^

Even mortality in our registry is lower than in other registries in the comparable period. This is explained by the center's HF‐focused care with a greater tendency to adhere to the recommended procedures, titrating recommended medications.^21^

According to the Long‐Term Survival Outcomes in Patients With Heart Failure meta‐analysis, diabetes was associated with a higher risk of all‐cause death, CV death, and hospitalization. Similarly, although borderline significantly, diabetes was a risk factor in our registry.^22^

Another study showed that a multiparametric or multibiomarker approach is the best method for prognosis stratification. In a long‐term chronic HF registry, patients with reduced EF were compared with multiparametric prognostic scores, and the MECKI (Metabolic Exercise test data combined with Cardiac and Kidney Indexes) score was superior to other tested scores.^23^ Regarding our registry, DM should also be included in the multibiomarker approach to make the prognosis more accurate.^24^, ^25^

The presence of DM as a prognostic indicator has already been shown in the AHEAD registry in AHF, where the prognosis of patients depended most on the five comorbidities reflecting the AHEAD score (Atrial fibrillation, low Hemoglobin level or anemia, Elderly; Abnormal renal function and D as Diabetes mellitus).^26^

In the FAR NHL registry, the male‐to‐female ratio was very high (80.3%), and even in other HF trials, males always predominated. In the Brno transplantation center, where one of the inclusion criteria for heart transplantation is age below 65 years, women of this age were much less represented than men in the HF group with ischemic heart disease etiology. In women, ischemic heart disease was more common at older ages. Similar proportions of men and women were observed in the ESC‐HFA Heart Failure Long‐Term Registry.^4^

## 5 CONCLUSION

According to the FAR NHL registry, patients with diabetes more often had comorbidities such as ischemic heart disease, hypertension, dyslipidemia, or renal impairment, higher uric acid, lower hemoglobin, and lower eGFR. These parameters are associated with higher NT‐proBNP values. Higher NT‐proBNP in patients with diabetes are explained by these factors, not the very presence of diabetes. The combination of diabetes and NT‐proBNP levels may help stratify the prognosis of patients with chronic HF when the NT‐proBNP level is low; nevertheless, high NT‐proBNP is in itself a strong predictor of events that the addition of DM will no longer improve the stratification of patient prognosis in this group. This may help to better manage and personalize care for each HF patient.

## 6 LIMITATIONS

The FAR NHL registry is a Czech multicenter prospective registry that may not resemble the heterogeneity of the European patient population. There may have been differences between the three different hospitals; in particular, St. Anne's University Hospital treats patients before OHT, so there were more patients with severe HF than in the other two centers. This may have affected the outcome and prediction. Basic laboratory measurements including NT‐proBNP were not performed in the core laboratory and are subject to interlaboratory variability. All patients were Caucasian, reflecting the population of the Czech Republic. This limits the generalizability of the models.

Inclusion in the FAR NHL registry was before the discovery of the cardioprotective effects of SGLT2i and its positive impact on the prognosis of patients with HF. Thus, patients were not receiving these drugs at the time of registry inclusion.

Patients were included only from those admitted to cardiology departments or examined in cardiology outpatient clinics; therefore, patients with HF examined in other departments, such as the emergency department or other clinics and departments, were not considered.

Finally, because of the short follow‐up of the registry, long‐term changes were not much expressed. We plan to collect further follow‐up data.

## AUTHOR CONTRIBUTIONS

All authors had full access to all the data in the study and take responsibility for the integrity of the data and the accuracy of the data analysis. Labr K., Spinarova M., and Spinarova L. conceived and designed the meta‐analysis. Labr K. drafted the manuscript. Spinarova M. and Spinarova L. acquired the data and co‐revised the manuscript. All authors read and approved of the final manuscript. The main author of the article is Labr K., the correspondence was arranged by Spinarova M., and other co‐authors contributed equally to the manuscript.

## FUNDING INFORMATION

No financial conflict or other relationship for each author to be declared.

## CONFLICT OF INTEREST STATEMENT

The authors declare no conflicts of interest.
